# Management and Diagnosis of Infertility and Recurrent Abortions in Obstetrics and Gynecology

**DOI:** 10.3390/medicina62101955

**Published:** 2026-10-10

**Authors:** Sofoklis Stavros, Anastasios Potiris, Peter Drakakis

**Affiliations:** Third Department of Obstetrics and Gynecology, University General Hospital “ATTIKON”, Medical School, National and Kapodistrian University of Athens, 12462 Athens, Greece

Infertility and recurrent pregnancy loss (RPL) remain major challenges in reproductive medicine because of their complex and frequently multifactorial etiology. Genetic, anatomical, hormonal, immunological, infectious, environmental, and lifestyle-related factors may contribute to both conditions, often through overlapping biological pathways [1,2,3]. Against this background, this Special Issue, entitled “Management and Diagnosis of Infertility and Recurrent Abortions in Obstetrics and Gynecology”, of *Medicina* aimed to advance the understanding of the genetic and molecular basis of RPL and infertility, explore novel diagnostic and therapeutic strategies, address male and female determinants of assisted reproductive technology (ART) outcomes, and examine early-pregnancy complications. A particular strength of this collection is the inclusion of five original research articles, four narrative and systematic reviews, and a clinically informative case report. Together, the ten contributions illustrate the breadth of contemporary reproductive medicine, extending from molecular mechanisms and diagnostic refinement to individualized treatment and patient-centered care.

Regarding the multifactorial nature of RPL, Uța et al. reviewed alloimmune mechanisms at the maternal–fetal interface, highlighting the coordinated roles of natural killer cells; T-cell subsets; regulatory T cells, B cells, and cytokines; and trophoblast-derived signals in establishing maternal tolerance. Pregnancy requires a carefully regulated balance among the inflammatory mechanisms necessary for implantation and vascular remodeling and the immunological pathways that permit tolerance of the semi-allogeneic conceptus. The authors also discussed immunomodulatory approaches, including intravenous immunoglobulin, glucocorticoids, and tumor necrosis factor-α inhibitors, acknowledging the limitations and uncertainties surrounding their clinical application [4].

The paternal contribution to pregnancy loss was addressed by Kaltsas et al., who broadened the traditional female-centered framework of RPL. They examined oxidative stress, sperm DNA fragmentation, chromosomal abnormalities, epigenetic alterations, paternal age, general health, lifestyle, environmental exposures, and emerging proteomic biomarkers as potential determinants of pregnancy viability [5]. This perspective is particularly relevant because evaluations recurrent loss have predominantly focused on maternal factors. The increasing recognition of the importance of sperm genomic and epigenomic integrity suggests that RPL should more appropriately be considered a disorder of the reproductive couple rather than exclusively of the female partner.

Reinforcing the findings of the prior study, Jašinskienė and Čaplinskienė assessed the epidemiological patterns of male infertility in Lithuania and evaluated oxidative stress through measuring oxidation-reduction potential (ORP). Among 261 men undergoing ORP assessment, elevated oxidative stress was observed in 65.1%, including 48.5% of men with normozoospermia. The authors further found that incorporating oxidative stress assessment could reclassify some patients previously considered as having unexplained infertility within the framework of male oxidative stress infertility [6]. These findings suggest a complementary role for redox assessment while emphasizing that such biomarkers require further standardization and validation before routine clinical implementation.

The importance of modifiable determinants of reproductive success was explored in another clinical study by Micić et al. In a cohort of 501 infertile couples undergoing ART, the authors assessed occupational factors, smoking, alcohol consumption, nutritional habits, supplement use, physical activity, and general health characteristics. Clinical pregnancy was achieved in 22.2% of the study population. Younger age and the use of vitamins, minerals, and trace elements were associated with pregnancy among women, whereas younger age and absence of chronic illness were important factors among men. When couples were jointly considered, supplement use by both partners was associated with higher chances of clinical pregnancy [7]. These observational associations cannot establish causality; nevertheless, the study highlights the value of evaluating lifestyle and general health at the couple level and provides a rationale for prospective studies examining modifiable determinants of ART outcomes.

Anatomical factors are other important components of infertility and RPL. Petrovic et al. reviewed congenital anomalies affecting both the female and male reproductive systems, including Mullerian duct abnormalities, ovarian developmental disorders, cryptorchidism, anorchia, hypospadias, and disorders affecting ejaculation. Reproductive consequences considerably vary according to the nature and severity of the underlying abnormality. The authors emphasize early recognition, appropriate reproductive counseling, and multidisciplinary individualized management while noting that the clinical value of early diagnosis is condition-dependent [8].

Endometrial integrity and regeneration are similarly fundamental to reproductive success. Genco et al. investigated the effects of hyaluronic acid, methylprednisolone, and their combination in an experimentally induced rat model of Asherman syndrome. Hyaluronic acid increased uterine lumen diameter and vascularization, whereas methylprednisolone increased gland number and uterine wall thickness while reducing inflammation and fibrosis. The combination produced generally more favorable histopathological findings than either treatment alone [9]. Although these findings remain preclinical, the study provides a useful mechanistic framework in which endometrial restoration is approached through the simultaneous modulation of inflammation, fibrosis, angiogenesis, and tissue repair. Extrapolating these findings will require human studies incorporating clinically meaningful reproductive outcomes.

Infectious disease and especially chlamydial infection were addressed in the cohort study by Tošić-Pajić et al. The authors evaluated 225 sexually active individuals and assessed combinations of direct immunofluorescence, rapid immunochromatographic testing, and serological assays for the detection of *Chlamydia trachomatis*, using RT-PCR as the reference method. Their results show that strategies based on “two or more positive tests” or “any test positive” did not increase overall diagnostic efficiency compared with that achieved with a single “rapid test”. Certain combinations, particularly rapid testing with IgA measurement, were highly specific, although molecular nucleic acid amplification testing remains the preferred diagnostic approach, when available [10]. Beyond the specific findings, the authors stress that adding diagnostic tests does not necessarily improve diagnostic performance, and new strategies should demonstrate meaningful value before implementation.

Individualizing strategies for triggering final oocyte maturation in patients with polycystic ovary syndrome undergoing IVF was examined in a retrospective cohort study by Yilmaz et al. The authors included 267 IVF cycles and compared gonadotropin-releasing hormone agonist (GnRHa) and human chorionic gonadotropin (hCG) triggering. The numbers of retrieved oocytes, mature oocytes, fertilized oocytes, and good-quality embryos were higher with GnRHa-triggered cycles followed by frozen embryo transfer. Despite these differences, clinical pregnancy rates were comparable among GnRHa-triggered frozen transfer, hCG-triggered frozen transfer, and hCG-triggered fresh transfer groups [11]. These findings support increasing the emphasis on individualized final oocyte maturation strategies in patients with PCOS, in whom ovarian response and the risk of ovarian hyperstimulation syndrome must be balanced against embryo yield, luteal physiology, and the planned transfer strategy. Importantly, optimization should not be defined according solely to the number of oocytes retrieved but also to the overall safety and reproductive efficiency of the treatment cycle.

Early pregnancy management represents another dimension of this Special Issue. Stavros et al. describe a rare case of severe methotrexate toxicity following ultrasound-guided intragestational administration for a caesarean scar pregnancy. Despite the local administration of a relatively low dose, the patient developed oral and cutaneous manifestations, febrile neutropenia, and ultimately severe pancytopenia secondary to bone marrow suppression, requiring intensive treatment before recovery [12]. The report provides a clinical reminder that the local administration of methotrexate does not eliminate the possibility of systemic toxicity and that careful surveillance remains essential.

Papalois et al. systematically reviewed the psychological experiences of pregnancy following organ transplantation. From a qualitative synthesis, the authors identified four principal themes: perception of pregnancy after transplantation, concerns regarding maternal physical health, concerns regarding fetal health, and emotional burden and coping strategies [13]. Although this population differs from conventional infertility cohorts, the findings of the study address one of the core objectives of this Special Issue that reproductive care should incorporate psychological well-being alongside clinical outcomes.

Taken together, the publications in this Special Issue highlight several priorities that will likely shape the future of reproductive medicine. The ten publications provide complementary perspectives on endometrial regeneration, reproductive infection diagnostics, male oxidative stress, individualized ovarian triggering, occupational and lifestyle factors influencing ART outcomes, immunological mechanisms, paternal contributions, reproductive anatomy, early-pregnancy complications, and psychological well-being. Collectively, these studies emphasize that infertility and recurrent pregnancy loss are heterogeneous and frequently multifactorial conditions that are best approached through integrated, couple-centered, and multidisciplinary care [14].

Further progress will depend on the prospective and multicenter validation of emerging biomarkers and diagnostic tools, the standardization of methodologies, and the use of clinically meaningful reproductive outcomes [15,16]. Increased attention should also be directed toward the interaction between the male and female determinants of reproductive success, the identification of modifiable lifestyle and environmental factors, and the development of treatment strategies tailored to individual biological and clinical characteristics [17,18,19]. Simultaneously, psychological well-being and patient-reported outcomes should be more consistently incorporated into reproductive research and clinical practice, reflecting the substantial emotional burden associated with infertility, recurrent pregnancy loss, and medically complex pregnancies [20,21].

The diversity of topics addressed in this Special Issue reflects the breadth and complexity of modern reproductive medicine. Rather than providing a definitive endpoint, this collection identifies areas in which further mechanistic and clinical investigations remain necessary and highlights the value of integrating biological, diagnostic, therapeutic, and patient-centered perspectives. In view of the continuing scientific interest in these topics, Volume II of “Management and Diagnosis of Infertility and Recurrent Abortions in Obstetrics and Gynecology” is currently open for submissions, providing an opportunity to further expand the evidence base and incorporate new developments in the diagnosis, treatment, and multidisciplinary management of infertility and recurrent pregnancy loss. We hope that the present collection, together with the forthcoming contributions to Volume II, will encourage continued collaboration and help advance more precise, individualized, and patient-centered reproductive care.

## Data Availability

No new data were created or analyzed in this study. Data sharing is not applicable to this article.
